# Interaction between clock genes, melatonin and cardiovascular outcomes from ICU patients

**DOI:** 10.1186/s40635-025-00730-2

**Published:** 2025-02-17

**Authors:** Jose M. Jiménez-Pastor, Ignacio Morales-Cané, Francisco J. Rodríguez-Cortés, Luna López-Coleto, Rocío Valverde-León, Pedro Arévalo-Buitrago, María J. Medina-Valverde, Carmen De la Fuente-Martos, Darío Acuña-Castroviejo, Miguel Meira e Cruz, Raúl M. Luque, André Sarmento-Cabral, Pablo J. López-Soto

**Affiliations:** 1 GC31 Group, Maimonides Institute of Biomedical Research of Córdoba (IMIBIC), Cordoba, Spain; 2 Department of Nursing, Pharmacology and Physiotherapy, University of Córdoba, Córdoba, Spain; 3 Department of Nursing, Reina Sofia University Hospital, 14004 Cordoba, Spain; 4 Department of Intensive Care Medicine, Reina Sofia University Hospital, 14004 Cordoba, Spain; 5 Centro de Investigación Biomédica, Departamento de Fisiología, Facultad de Medicina, Parque Tecnológico de Ciencias de la Salud, Universidad de Granada, 18016 Granada, Spain; 6 Sleep Unit, Centro Cardiovascular da Universidade de Lisboa, Lisbon School of Medicine, Lisbon, Portugal; 7 Centro Europeu do Sono, Lisbon, Portugal; 8 GC27 Group, Maimonides Institute of Biomedical Research of Córdoba (IMIBIC), Córdoba, Spain; 9 Department of Cell Biology, Physiology and Immunology, University of Córdoba, Córdoba, Spain; 10 Reina Sofía University Hospital (HURS), Córdoba, Spain; 11 CIBER Pathophysiology of Obesity and Nutrition (CIBERobn), Córdoba, Spain

**Keywords:** Intensive care unit, Melatonin, Clock genes, Circadian rhythms, Disruption, Cardiovascular, Critical illness

## Abstract

**Background:**

Circadian rhythms, driven by biological clocks, help organisms align their physiological functions with environmental changes, promoting homeostasis. The central clock in the suprachiasmatic nucleus coordinates peripheral clocks via neurohumoral feedback involving proteins like CLOCK, BMAL1, CRY 1/2, and PER 1–3. In the ICU, these circadian processes often face disruptions from constant lighting, noise, and irregular sleep–wake cycles, impairing sleep quality and worsening stress responses. These disruptions can lead to adverse clinical effects, including higher cardiovascular complication rates. This study examines how ICU stays affect circadian rhythm regulators and their association with cardiovascular outcomes.

**Results:**

Significant differences were identified in melatonin levels and the expression of BMAL1, PER1, RORA, and NR1D1 between ICU stays of ≤7 days and >7 days. The APACHE-II severity scale influenced melatonin and the expression of CLOCK, PER2, CRY2, and RORA. Nonlinear relationships were observed between melatonin, clock genes, heart rate, and blood pressure (systolic and diastolic). In certain groups, molecular and physiological data showed correlations exceeding 90%.

**Conclusions:**

These findings highlight a robust association between circadian disruption, as measured by melatonin and clock genes, and cardiovascular physiological rhythms in ICU patients.

**Supplementary Information:**

The online version contains supplementary material available at 10.1186/s40635-025-00730-2.

## Background

Circadian rhythms, characterized by biological cycles with an approximately 24-h period, have been identified across all mammalian cell types. The primary function of cellular biological clocks is to orchestrate circadian rhythms, allowing organisms to anticipate and adapt to various demands proactively [1]. In humans, the central circadian clock is situated in the suprachiasmatic nucleus (SCN). Light-regulated, it governs peripheral clocks through neurohumoral modulation [2]. A transcriptional–translational feedback loop that includes transcriptional activators CLOCK and BMAL1 and repressor CRY 1/2 and PER 1–3, which oversee the daily transcription of clock genes [3]. It has been demonstrated that circadian disruption in humans is associated with an increased risk of heart disease and worse outcomes following cardiac injury [4]. Emerging evidence suggests that the Clock gene regulates mitochondrial health by transcriptionally controlling mitochondrial dynamics, bioenergetics, and quality control processes in cardiac myocytes. A link between Clock gene activity and the survival of cardiac cells has also been discovered. Moreover, the loss of Clock gene activity, both in vitro and in vivo, results in increased cardiac injury and ventricular dysfunction due to impaired activation of autophagy/mitophagy, mitochondrial bioenergetics, and mitochondrial dynamics [5]. In addition, the loss of BMAL1 has been linked to cardiac developmental abnormalities and cardiac hypertrophy [6]. In fact, circadian misalignment, which is commonly observed with jet lag or shift work, has been associated with metabolic dysregulation [7] and a higher incidence of cardiac injury following myocardial infarction [8].

In this context, circadian rhythms regulate the onset, severity, and outcomes of many cardiovascular diseases, including myocardial infarction, diabetic cardiomyopathy, doxorubicin-induced cardiotoxicity, and heart failure [9]_._

Rodent models have demonstrated that the circadian clock plays an integral role in regulating blood pressure, diabetes, and metabolism. Clock genes such as BMAL1, CLOCK, Per1, Per2, Cry1, and Cry2 have been implicated in various diseases, including hypertension, diabetes, and obesity. CLOCK, BMAL1, and Per2 play key roles in the heart and vascular system. Per1 and CLOCK have critical functions in sodium reabsorption in the kidney [10].

BMAL1 knockout (KO) mice exhibit lower blood pressure during the active phase [11], resulting in the elimination of circadian variation in blood pressure. Part of this reduced blood pressure phenotype has been attributed to changes in the vascular system of these mice. BMAL1 KO mice show increased endothelial dysfunction due to impaired nitric oxide signaling [12]. Recent studies have shown that CLOCK plays an important role in regulating renal function and blood pressure. CLOCK KO mice are hypotensive compared to wild-type mice, exhibit mild diabetes insipidus, and excrete more sodium in their urine [13]. CLOCK KO mice have reduced blood pressure, similar to BMAL1 KO mice.

Per1 has been shown to play a novel role in regulating renal sodium reabsorption and blood pressure. Per1 regulates both basal and aldosterone-induced regulation of the alpha subunit of the epithelial sodium channel in renal collecting duct cells. A recent human study showed that Per1 mRNA expression was significantly increased in the renal medulla of hypertensive patients compared to normotensive controls [10], suggesting a role for Per1 in renal blood pressure regulation in humans. Per2 mutant mice have reduced diastolic pressure over 24 h, a slight increase in heart rate, and a diminished daytime blood pressure dip [14].

Cry1 and Cry2 have been identified as major circadian repressors. Mice with Cry1/2 knockout (KO) exhibit salt-sensitive hypertension, associated with elevated plasma aldosterone levels and higher levels of 3-beta-hydroxysteroid dehydrogenase–isomerase, an adrenal-specific enzyme involved in aldosterone synthesis [10].

Melatonin, produced from serotonin in the pineal gland, regulates sleep–wake cycles, circadian rhythms, immune function, and pituitary and adrenal hormones [15, 16]. It affects thermoregulation and cardiovascular centres, aiding in the physiological oscillation of blood pressure (BP) and heart rate (HR).

Melatonin exerts various actions, such as antioxidant effects, sympatholytic action, interference with variable humoral systems, and metabolic actions. These effects reduce BP and HR, improving endothelial function and exerting an antifibrotic impact on the heart, vessels, and potentially cardiovascular protection [17]. The extraordinary antioxidant potential reduces oxidative stress and enhances nitric oxide’s bioavailability, thereby exerting cardiovascular protection through vasodilation, hypotension, and inhibition of pathological growth [18].

The lack of synchronized rhythmic processes, mainly those showing a circadian basis, like sleep, heart rate, and arterial pressure, often leads to several organic challenges eventually associated with adverse outcomes. Meanwhile, the hostile Intensive Care Unit (ICU) environment is typically linked to a negative impact on both the circadian timing system and sleep regulation centres, which has important implications in the recovery of critically ill patients [19, 20].

This study aimed to explore the impact of short- and long-term stages in ICU on the gene expression of primary circadian rhythm regulators RORA, NR1D1, PER1, PER2, CRY1, CRY2, BMAL1, CLOCK, and melatonin.

## Methods

### Ethical considerations

This study was conducted according to the guidelines of the Declaration of Helsinki and approved by the Reina Sofia University Hospital Ethics Committee (Act nº 277; ref. 3878).

Patients older than 18 years were provided with an information sheet before inclusion in the study, and informed consent was obtained from all participants. If patients could not consent due to their clinical condition, consent was obtained from their families.

### Characteristics of the cohort of patients included in the study

A prospective study involved patients with cardiovascular pathologies admitted to the ICU of a third-level hospital in southern Spain between October 2020 and August 2021. Participants were required to be aged over 18 years and be treated in ICU for at least 48 h. Pregnant women and patients admitted to restricted access modules were excluded. Of the patients admitted to the ICU, 21 were selected for this study. All with mechanical ventilation and a mean age of 60.48 ± 20.50 years, 71.43% of them males. The primary reason for patients’ admission to the ICU was cardiovascular surgery (47.62%) (Table 1). We have no evidence that any of the patients included in the study exhibited neurological deterioration during the assessment study. The drug used for the sedation of patients was propofol.

**Table 1:** Clinical variables of the patients with cardiovascular disease admitted to the ICU

Age	60.48 (20.50)
Gender	
Male	15 (71.43%)
Female	6 (28.57%)
Clinical diagnosis	
Cardiovascular surgery	10 (47.62%)
ST-elevation myocardial infarction (STEMI)	5 (23.81%)
Non-rheumatic mitral valve disease	1 (4.76%)
Out-of-hospital cardiac arrest	1 (4.76%)
Cardiogenic shock + heart transplant	4 (19.05%)
HR (bpm)	94.05 (1.44)
SBP (mmHg)	115.7 (2.52)
DBP (mmHg)	61.60 (1.54)
Mean blood pressure (mmHg)	79.64 (1.61)
SpO2 (%)	98.09 (0.41)
Mechanical ventilation	21 (100%)
ICU Length of stay	29.76 (16.78)
Hospital discharge (survival)	11 (52.38%)
APACHE-II	10.50 (1.17)
RASS (Median + Min, Max)	−2.00 (−5, 2)
Patient with pacemaker (%)	2 (9.52%)
Sedated patient (%)	7 (33.33%)
Patients on vasopressors or inotropes (%)	14 (66.66%)

Data presented as number of subjects and percentage (%) or Mean (SD). *HR* heart rate, *SBP* systolic blood pressure, *DBP* diastolic blood pressure, *ICU* intensive care unit, *RASS* Richmond Agitation Sedation Scale

### Variables recorded and patients follow-up during ICU stay

Physiological data on the patient’s well-being is monitored using a physiologic monitor, which records vital parameters such as heart rate, respiratory rate, oxygen saturation, and blood pressure. Data were collected every 15 min. The patient’s level of consciousness is assessed using the Glasgow Coma Scale. It also used the RASS scale to measure the patients’ sedation and agitation state. Finally, RASS is included in Table 1 instead of the Glasgow scale, because we did not have complete Glasgow data for certain patients.

### Blood sample collection and preparation

Blood samples were collected from ICU patients to obtain peripheral blood mononuclear cells (PBMCs) cells to analyse the gene expression of clock genes, specifically RORA, NR1D1, PER1, PER2, CRY1, CRY2, BMAL1, and CLOCK. Blood samples were collected at specific intervals over 48 h during patient monitoring. The collection times were consistent (specifically at 8:00. 13:00. 18:00, and 23:00). A Ficoll separation method was employed to isolate PBMCs using Lymphoprep™ Reagent. Then, RNA was isolated using the triazole–chloroform method. After extraction, RNA was quantified using a Thermo Fisher Scientific Inc® Nanodrop 2000 UV–Vis spectrophotometer. Complementary DNA (cDNA) was synthesized from one µg of RNA (independently of the RNA obtained in each sample to normalize the results) using the RevertAid First Strand cDNA Synthesis Kit® according to the manufacturer’s instructions. Finally, quantitative real-time PCR was conducted using SYBR Green qPCR Master Mix Agilent®, employing the Mx3000P Stratagene® Thermocycler. The results were normalized by calculating a ratio between the gene under study and ACTB as the reference gene.

### Quantification of 6-sulfatoxymelatonin in urine as a surrogate of melatonin levels in plasma

Melatonin rhythm profiles were analyzed using urine samples (accessed by measuring 6-sulfatoxymelatonin) every 2 h for 48 h. Samples underwent centrifugation at 3000 rpm for 10 min and then frozen at −20 °C until the determination of 6-sulfatoxymelatonin levels using an ELISA assay (TECAN–RE54031). The measurement of melatonin was conducted using urine samples, as this method allowed for the collection of multiple samples throughout the day without the need for an invasive procedure, such as blood extraction from the patient. Collecting blood every 2 h would not have been feasible, whereas this frequency was achievable with urinary melatonin measurements.

### Data analysis

Patient’s characteristics were assessed using descriptive statistics. Normality was tested using the Shapiro–Wilk test, and variance homogeneity was assessed using the Levene test. We utilized the two-way ANOVA test to compare mean values between two groups of quantitative variables.

Variability of physiological data, clock genes, and melatonin levels in ICU patients was established through Generalized Additive Mixed Models (GAMMs). GAMMs allow us to capture the non-linear effects of time on gene expression levels and vital parameters by utilizing smoothing splines. GAMMs are widely used for examining the variability in repeated measurements within and between subjects, incorporating random effects, and assessing non-linear effects of covariates on the response variable through smooth functions [21].

Fourier series were used to explore the potential rhythmic patterns of clock genes and melatonin. These series decompose periodic functions into the sum of simple oscillating functions, such as sines and cosines, making them particularly useful for approximating periodic functions [22]. This study applied the Fourier series to assess the periodicity of clock genes and melatonin. The periodic functions were obtained using Matlab R2023b software.

## Results

### Differences in melatonin levels and clock gene expression associated with length of stay in the ICU

**Fig. 1:**
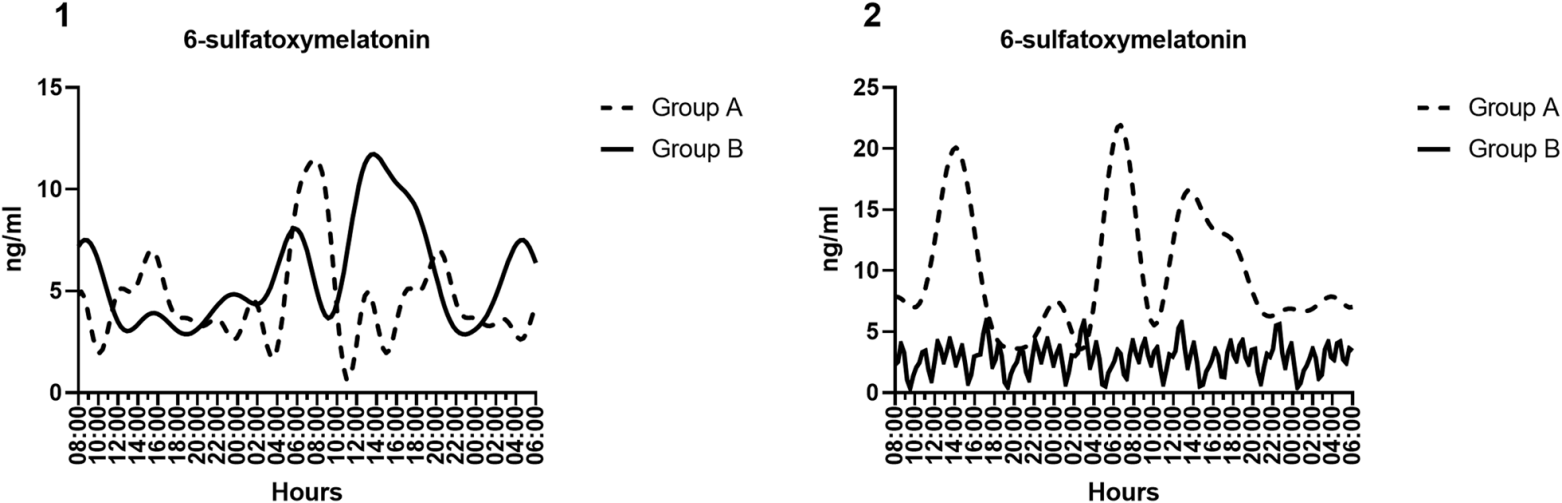
Representation of the Fourier model of 6-sulfatoxymelatonin detection in urine, as surrogate of plasma melatonin levels: **1** Group A (Short stay in ICU)—R-squared = 0.9821, Group B (Long stay in ICU)—R-squared = 0.9506; **2** Group A (APACHE-II score of 10 or lower)—R-squared = 0.9663, Group B (APACHE-II score higher than 10)—R-squared = 0.9189

Patients were divided into two groups: Patients who had a maximum of 7 days of stay in the ICU (short-stay group, *n* = 10) and patients who had been hospitalized in the ICU for more than 7 days (long-stay group, *n* = 11). A 1-week stay in the ICU was used as the time measure to divide patients into two groups, as it was considered a significant time unit. This division also yielded two homogeneous groups, taking into account the data dispersion. The expression of clock genes was analyzed using a two-way ANOVA, with the length of stay in the ICU and the time of day at which the samples were collected as the factors under study. Our data revealed a significant difference in melatonin levels between short- and long-stay groups (length of stay *p* = 0.0271; interaction of the two factors *p* = 0.0392). At a graphical level, we observe an irregular pattern in both groups (Fig. 1). Differences were found in BMAL1 (length of stay *p* = 0.0023, the time of day *p* = 0.0285), PER1 (length of stay *p* = 0.0361), RORA (interaction of the two factors *p* = 0.0085), and NR1D1 (length of stay *p* = 0.0361 and *p* = 0.0044, respectively) (Table 2).

**Table 2:** *p* values of the two-way ANOVA with length of stay and sample collection time as study factors

GENES/PROTEIN	Two-way ANOVA			Expression levels	
	Interaction	Time of collection	Length of stay	Short stay	Long stay
CLOCK	0.1542	0.3823	0.6338	1.39E−04 (2.36E−04)	1.01E−04 (9.76E−05)
BMAL1	0.0779	0.0285*	0.0023*	1.186E−07 (8.45E−08)	8.795E−07 (9.988E−07)
PER1	0.6832	0.9148	0.0361*	2.18E−02 (5.58E−03)	1.47E−02 (3.84E−03)
PER2	0.5641	0.1124	0.9637	1.28E−04 (7.72E−05)	1.27E−04 (5.13E−05)
CRY1	0.5851	0.5279	0.3861	1.80E−04 (1.14E−04)	6.44E−04 (1.38E−03)
CRY2	0.149	0.7993	0.5877	6.19E−04 (2.35E−04)	5.52E−04 (2.78E−04)
RORA	0.0085**	0.0046**	0.5286	2.36E−03 (2.38E−03)	1.98E−03 (1.52E−03)
NR1D1	0.1982	0.1025	0.0044**	9.08E−06 (8.53E−06)	1.90E−06 (9.16E−07)
MELATONIN	0.0392*	0.0982	0.0271**	4.53 (2.24)	5.78 (2.56)

Expression levels: clock genes (relative expression with respect to ACTB), melatonin (ng/ml), mean (SD), *p* value: *<0.05, **<0.01

### Differences in melatonin levels and clock gene expression associated with patient severity

Patients were divided into two groups: a low-APACHE group (APACHE ≤ 10, *n* = 9) and a high-APACHE group (APACHE > 10, *N* = 12). This division into two groups was based on the mean APACHE-II score of the patients included in the study. The two-way ANOVA shows significant values when studying melatonin levels (Severity *p* < 0.0001, collection time *p* = 0.0046, interaction between both factors *p* = 0.0062). Visually, the low-APACHE group exhibits a pattern with peaks at 14:00 and 06:00, while the high-APACHE group does not show a clear pattern (Fig. 1). CLOCK, PER2, CRY2, NR1D1 (APACHE-II factor, *p* = 0.0009, *p* = 0.0002, *p* = 0.003, and *p* = 0.0083, respectively) and RORA APACHE-II factor *p* = 0.048, interaction of both factors *p* = 0.0457) show significant values. BMAL1 showed differences when considering the time of day of sample collection (*p* = 0.0043) (Table 3). When comparing the two groups, there were significant differences in HR, systolic blood pressure (SBP), and diastolic blood pressure (DBP) (Supplementary Table 1).

**Table 3:** *p* values of the two-way ANOVA with APACHE-II and sample collection time as study factors

GENES/PROTEIN	Two-way ANOVA			Expression levels	
	Interaction	Time of collection	APACHE-II	Low-A	High-A
CLOCK	0.0881	0.4792	0.0009***	1.37E−04 (8.66E−05)	3.67E−05 (4.14E−05)
BMAL1	0.4612	0.0043**	0.1629	6.82E−07 (7.62E−07)	3.36E−07 (6.50E−07)
PER1	0.2221	0.8463	0.7549	2.15E−02 (9.97E−03)	2.00E−02 (8.02E−03)
PER2	0.0984	0.0914	0.0002***	3.29E−04 (2.37E−04)	9.46E−05 (3.66E−05)
CRY1	0.1323	0.1330	0.2168	3.42E−04 (2.60E−04)	1.10E−02 (3.04E−02)
CRY2	0.1402	0.1653	0.0003***	1.12E−03 (6.09E−04)	4.68E−04 (2.05E−04)
RORA	0.0457*	0.6304	0.0480*	3.52E−03 (1.69E−03)	2.05E−03 (1.82E−03)
NR1D1	0.1888	0.1839	0.0083**	8.79E−06 (8.12E−06)	2.06E−06 (1.21E−06)
MELATONIN	0.0062**	0.0046**	<0.0001***	9.70 (5.00)	3.03 (0.68)

Expression levels: clock genes (relative expression with respect to ACTB), melatonin (ng/ml), mean (SD), *p* value:*<0.05, **<0.01, ***<0.001

### Effect of melatonin and clock genes on the variability of cardiovascular variables

Using GAMMs, the effect of melatonin and clock genes on the variability of cardiovascular variables was studied. For each study variable, models included the most significant possible number of clock genes to explain as high a percentage as possible of the variability of the dependent variable.

#### Effect of ICU length of stay

Regarding HR, for the short-stay group in the ICU, a GAMM model was generated that includes all eight genes and melatonin, explaining 99.60% of the observed variability in HR, with an adjusted *R*-squared (*R*-sq(adj)) of 0.986. All independent variables included in the model were significant except for PER1 (*p* = 0.13), which was retained as its removal worsened the overall model. On the other hand, in the long-stay group, the best possible model includes melatonin, CLOCK, BMAL1, PER1, and RORA variables. This model explains 74.90% of the variability with an *R*-sq(adj) of 0.63. Melatonin is not significant in this model, but removing it from the model significantly worsens its performance.

In SBP, the model for the short-stay group explains 100% of the variability with an *R*-sq(adj) of 1. This model only excludes the variable PER2. The model explains 99.40% of the variability for the long-stay group with an *R*-sq(adj) of 0.971, including all variables.

Finally, the DBP was studied, obtaining a model in the short-stay group that explains 58.80% of the variability with an adjusted *R*-squared *R*-sq(adj) of 0.418. This model includes melatonin, CRY1, PER2, RORA, and NR1D1. On the other hand, the long-stay group presents a model that explains 91.40% of the variability with an *R*-sq(adj) of 0.797, including melatonin and all genes. However, CRY2 and PER2 do not have a significant *p* value (Table 4).

**Table 4:** *p* values of the independent variables in the GAMM models of patients grouped by length of stay in the ICU

	HR-A	HR-B	SBP-A	SBP-B	DBP-A	DBP-B
MELATONIN	0.0004	0.8847	0.0077	0.2587	0.7968	0.0216
CLOCK	0.0022	0.0009	0.0026	0.0028		0.0328
BMAL1	0.0081	0.0004	0.0062	0.0011		0.0062
CRY1	0.0018		0.0005	0.0062	0.0109	0.0053
PER1	0.1312	0.0009	0.0091	0.0391		0.0197
CRY2	0.0024		0.0105	0.0175		0.1783
PER2	0.0331			0.0012	0.0386	0.1050
RORA	0.0071	0.0003	0.0271	0.0005	0.0346	0.0196
NR1D1	0.0202		0.0169	0.0105	0.0356	0.0033
*R*-sq(adj)	0.986	0.630	1	0.971	0.418	0.797
Deviance exp	99.60%	74.90%	100.00%	99.40%	58.80%	91.40%

A (short stay), B (long stay), *R-sq(adj)* Adjusted R-squared, *Deviance exp* deviance explained, *HR* heart rate, *SBP* systolic blood pressure, *DBP* diastolic blood pressure

#### Effect of patient severity

When studying HR in the low-APACHE group, we obtain a model that explains 54.70% of the variability of this dependent variable with an *R*-squared *R*-sq(adj) of 0.497. This model only includes melatonin and NR1D1 as independent variables. In the case of the high-APACHE group, the model explains 50% of the variability in HR with an *R*-sq(adj) of 0.413. This model includes CRY1-2 and melatonin, although the latter does not show a significant value.

The model for the low-APACHE group explains 99.30% of the variability in SBP with an R-sq(adj) of 0.971. This model includes all independent variables except CRY1, although melatonin does not show a significant *p* value. The model for the other group explains 92.70% of the variability in SBP with an *R*-sq(adj) of 0.831. This group includes all variables except RORA and NR1D1, although of all included variables, only CLOCK, BMAL1, and CRY1 show significant values.

Finally, the DBP was studied, and in the low-APACHE group, an adequate model could not significantly explain variability. On the other hand, in the other group, the model explains 58.8% of the variability in DBP, with an *R*-sq(adj) of 0.499. This model includes melatonin, CLOCK, and NR1D1 (Table 5).

**Table 5:** *p* values of the independent variables in the GAMM models of patients grouped by APACHE-II score

	HR-A	HR-B	SBP-A	SBP-B	DBP-A	DBP-B
MELATONIN	0.0069	0.0844	0.9262	0.7144	0.9552	0.7806
CLOCK			0.0008	0.0076		0.0240
BMAL1			0.0049	0.0009		
CRY1		0.0445		0.0007	0.0109	
PER1			0.0010	0.1099		
CRY2		0.0183	0.0009	0.1927		
PER2			0.0005	0.5592	0.0827	
RORA			0.0039		0.0413	
NR1D1	0.0080		0.0102			0.0010
*R*-sq(adj)	0.497	0.413	0.971	0.831	0.220	0.499
Deviance exp	54.70%	50.00%	99.30%	92.70%	39.00%	58.8%

A (low APACHE-II), B (high APACHE-II), *R-sq(adj)* adjusted *R*-squared, *Deviance exp* deviance explained, *HR* heart rate, SBP systolic blood pressure, DBP diastolic blood pressure

## Discussion

A primary outcome from this study is the affirmative finding that significant changes in the expression of some clock genes and temporal secretion of melatonin occur in critically ill patients admitted to the ICU.

Administered in healthy men, melatonin decreased heart rate and blood pressure, increased heart rate variability, and reduced plasma norepinephrine and dopamine were observed [23]. In normotensive and hypertensive elderly volunteers, the administration of melatonin for 2 weeks induced a reduction in systolic and diastolic blood pressure and an attenuation of the increase in heart rate [24]. Similarly, patients with postural tachycardia syndrome demonstrated that melatonin significantly reduced heart rate compared to placebo [25]. Low melatonin levels may contribute to a detrimental metabolic profile and limited success of beta-blockers in hypertensive patients with elevated heart rate [17].

Numerous studies have examined the role of circadian clocks using rodent models with mutations in the null central clock gene. Male mice with global BMAL1 KO show blood pressure that does not increase during the active period, resulting in lower overall BP over 24 h and loss of diurnal BP rhythm [26]. It has been demonstrated that BMAL1 in smooth muscle is essential for hourly variations in phenylephrine- and serotonin-induced vasoconstriction of renal and mesenteric arteries. It is believed to reflect the attenuated BP rhythm in male BMAL1 KO smooth muscle-specific mice [27]. Adipocyte-specific BMAL1 KO male mice showed reduced blood pressure during the day, resulting in an extreme dipping phenotype [28].

Several circadian clock genes in the adrenal gland, such as BMAL1, PER2, and CRY1, showed altered patterns for spontaneously hypertensive rats compared to control ones. The serum corticosterone and aldosterone levels were also abnormal. Both steroids have been implicated in the rhythm of blood pressure [26].

All the points above lead us to the certainty that melatonin and clock genes have a direct effect, especially highlighting BMAL1, on HR and BP. This is consistent with the results obtained in our GAMM models, where we studied the direct relationship between melatonin and clock genes and heart rate and BP. Specifically, in the GAMM models performed with patients grouped by their ICU stay time, BMAL1 appears significantly in all models of heart rate, SBP, and DBP (except in the DBP model in the short-stay group). Other genes, such as CLOCK, CRY1, PER1, RORA, and NR1D1, also appear significantly in the GAMM models conducted. These models explain a high percentage of SBP, DBP, and HR variability because of melatonin and clock genes on these three dependent variables. In the case of melatonin, which is included in all models considering it a key factor (demonstrated by the fact that removing it from the model results in a loss of explanatory percentage of variability), we observe that it presents substantial values in both HR and SBP models in the short-stay group, as well as in the DBP model in the long-stay group. In the GAMM models performed on patients grouped by their APACHE-II score, we only found models with high predictive reliability in the SBP models. In the rest of the models, we obtained percentages around 50% with R-squared values below 0.5. The fact that the models performed in groups of patients classified by their APACHE score show poorer data in explaining the implication of melatonin and clock genes on cardiovascular outcomes compared to the models performed in patients grouped by their ICU stay time could be due to more s heterogeneity in patients, making it challenging to obtain better-fitted GAMM models. This can be interpreted to mean that if we group patients by their APACHE score, we may obtain groups, where the heterogeneity of the patients is more significant than if we group them by their ICU stay time. This indicates that the "ICU stay time" factor is more effective in grouping patients homogeneously concerning their circadian state than their APACHE-II scores.

The relationship between the circadian rhythm and immunology has been demonstrated in both animal models and healthy humans, and at the molecular level, multiple immune cells show a circadian rhythm with high expression of regulatory clock genes, including CLOCK, BMAL, PER1-3, and CRY1-2 [29]. Along with this data, a previous study from our research group found high noise levels in a cardiovascular ICU. It established a relationship between noise, heart rate, respiratory rate, and the Glasgow Coma Scale. Specifically, we observed an association between increasing noise levels, heart rate, respiratory rate, and the Glasgow Coma Scale simultaneously, indicating that ICU environmental conditions influence certain variables related to patients’ physiological status [21]. Other studies have also linked blue light with a significant decrease in the concentration of aMT6s during the daytime and nighttime and the loss of its circadian rhythm [30]. A temporal association was also observed in a case series of 11 patients with primary neurological injury between the disruption of clock genes and ICU length of stay. Initially, at the time of admission, the circadian rhythm of clock genes (CLOCK, BMAL-1, CRY1, and PER2) was preserved; however, after 1 week, a disruption of the rhythm was observed [11]. All of this is consistent with our results, in which we found significant differences in melatonin levels and expression of several clock genes (BMAL1, PER1, RORA, and NR1D1) when comparing patients with a short ICU stay with patients who had a more than a week ICU length of stay. These results, along with the other studies mentioned above, confirm that ICU stay disrupts patients’ circadian rhythms, implying a potential worsening of the patient due to the involvement of clock genes in metabolic, cardiovascular, and immunological processes.

On the other hand, when comparing the expression of clock genes and plasma cytokines in healthy individuals with septic and non-septic ICU patients, it was demonstrated that the rhythm of clock genes was attenuated in septic patients, with higher innate immune and oxidative stress responses associated [31]. In our case, we found significant differences in melatonin levels and expression of several clock genes (CLOCK, BMAL1, PER2, CRY2, RORA, and NR1D1) when comparing patients with low APACHE-II scores and patients with high APACHE-II scores, therefore, suggesting an association between the individual circadian status and the severity of the patient condition.

Regarding the changes in melatonin patterns (Fig. 1), in the long-stay group, there is a delay in the maximum melatonin peak compared to the short-stay group. This may be due to the differences in clock gene expression observed between these groups (Table 2) and may also be associated with the continuous effect of blue light mentioned earlier. While in the low and high APACHE groups, a pattern with several peaks in the low APACHE group was observed, which could be explained by the effect of blue light or the fact that 70% of these patients were exposed to several hours of surgery in the days before the collection data. A complete loss of rhythmicity and a decrease in average melatonin levels compared to the low APACHE group (Table 4) were observed in the high APACHE group. This could be associated with the changes in clock genes observed between both groups (Table 3) and higher severity of the patient’s condition, along with the changes in HR, DBP, and SBP compared to the low APACHE group (Supplementary Table 1).

Various studies focused on restoring circadian rhythmicity have investigated potential therapies, including modifying external environments to align with natural light–dark cycles, administering melatonin, and adjusting feeding schedules. The use of melatonin in ICU patients seems to enhance and improve sleep quality and prevent or reduce the risk of delirium [32].

## Conclusions

Our results suggest that the regulators of the circadian timing system, which is the body’s internal clock governing the sleep–wake cycle and other physiological processes, are significantly associated with the health status of critically ill patients in an Intensive Care Unit (ICU). This impact is especially notable regarding cardiovascular parameters. In addition, our findings indicate that both the length of stay in the ICU and patient severity induce changes in the expression levels of clock genes as well as in blood melatonin levels (measured via its urinary metabolite), which may directly affect the patient’s health status.

Despite the number of participants included in the study may seem small and from a single-center, taking in account the high heterogeneity of the patients admitted to the ICU and the different action protocols between multiple centers, we took advantage of working with a single Andalusian reference Hospital, to carefully follow-up the patients. In addition, data on each patient’s physiological constants are collected every 15 min, resulting in 360 data points per physiological variable measured over 24 h. Thus, although *n* = 21 may seem relatively small, we work with a large volume of data that strengthens the analyses, especially in the context of Generalized Additive Mixed Models (GAMM). Therefore, our results underscore the need for broader studies to draw more generalized conclusions and to recognize the context-specific insights this study offers into ICU patient monitoring and care management. Nevertheless, this study may have significant implications for patients’ care and treatment, where the patient’s circadian state could serve as a predictive marker of the patient’s evolution.

In addition, this study highlights the importance of disrupting circadian rhythms in ICU patients. Thus, it sets new foundations for considering circadian state modulation as a treatment, through interventions in the patient’s environmental conditions or using melatonin, to improve their circadian rhythms. This could enhance the patients’ cardiovascular variables and expedite recovery, potentially representing a significant advancement in critical care medicine.

## Supplementary Information

Additional file 1.

## Data Availability

All data generated or analysed during this study are included in this published article [and its supplementary information files]. The data sets used and/or analysed during the current study are available from the corresponding author on reasonable request.

## Abbreviations

APACHE II: Acute physiology and chronic health evaluation II
BP: Blood pressure
cDNA: Complementary DNA
DBP: Diastolic blood pressure
GAMMs: Generalized additive mixed models
HR: Heart rate
ICU: Intensive care unit
PCR: Polymerase chain reaction
RASS: Richmond Agitation Sedation Scale
SBP: Systolic blood pressure
SCN: Suprachiasmatic nucleus
STEMI: ST-elevation myocardial infarction
