# Supplementary material for: Interaction between clock genes, melatonin and cardiovascular outcomes from ICU patients
Source: Intensive Care Med Exp. 2025 Feb 17;13:19. doi: 10.1186/s40635-025-00730-2 (PMC11832861; doi:10.1186/s40635-025-00730-2)
Supplement: Supplementary file 1 — Additional file 1. [file 40635_2025_730_MOESM1_ESM.docx]

**Supplementary information**

***Supplementary Table 1. Comparison between groups of cardiovascular variables.***

|  | **Length of stay** | | | **APACHE-II score** | | |
| --- | --- | --- | --- | --- | --- | --- |
|  | **Short-Stay** | **Long-Stay** | **T-test** | **Low-APACHE** | **High-APACHE** | **T-test** |
| **HR** | 94,00 (1,90) | 94,11 (2,07) | 0,8039 | 103,00 (2,05) | 86,28 (2,35) | **<0,0001** |
| **SBP** | 115,90 (4,11) | 115,60 (3,27) | 0,6734 | 111,30 (4,69) | 119,60 (4,62) | **<0,0001** |
| **DBP** | 62,12 (2,87) | 61,25 (2,32) | 0,1566 | 64,16 (2,20) | 59,46 (1,88) | **<0,0001** |

*HR - Heart rate; SBP - Systolic blood pressure; DBP - Diastolic blood pressure*

**Supplementary Table 2. Specific primers for human transcripts analysed by qPCR in this study.**

| **Gene Name** | **Description** | **Forward primer** | **Reverse primer** | **NCBI Reference Sequence** | **Product length** |
| --- | --- | --- | --- | --- | --- |
| ***ACTB*** | Homo sapiens actin beta | ACTCTTCCAGCCTTCCTTCCT | CAGTGATCTCCTTCTGCATCCT | NM_001101.5 | 176 |
| ***CLOCK*** | Homo sapiens clock circadian regulator | GGCATGTCCCAGTTTCAGTT | ACCCAGGATTTGATTGTTGC | NM_001267843.2 | 193 |
|  |  |  |  | NM_004898.4 |  |
| ***BMAL1*** | Homo sapiens basic helix-loop-helix ARNT like 1 | GTAACCTCAGCTGCCTCGTC | TAGCTGTTGCCCTCTGGTCT | Transcript variant 1 to 22 | 153 |
|  |  |  |  | NM_001351813.2 |  |
|  |  |  |  | NM_001351811.2 |  |
|  |  |  |  | NM_001351823.2 |  |
| ***PER1*** | Homo sapiens period circadian regulator 1 | AGTGGCTGTCTCCTTCCTGA | ACCTCCTCTGATACGGCAGA | NM_002616.3 | 250 |
| ***PER2*** | Homo sapiens period circadian regulator 2 | CCGGAGTTAGAGATGGTGGA | TGCTCCTCCTTCTGTGTGTG | NM_022817.3 | 161 |
| ***CRY1*** | Homo sapiens cryptochrome circadian regulator 1 | TTGCTTGATGCAGATTGGAG | TTTTGCAGGGAAGCCTCTTA | Transcript variant 1 to 14 | 174 |
| ***CRY2*** | Homo sapiens cryptochrome circadian regulator 2 | GTCCTGCAGTGCTTTCTTCC | TCCACACCAATGATGCACTT | NM_001127457.3 | 195 |
|  |  |  |  | NM_021117.5 |  |
| ***NR1D1*** | Homo sapiens nuclear receptor subfamily 1 group D member 1 | CTGCTGGCATGTCCTATGAA | GCTGAGAAAGGTCACGGAAG | NM_021724.5 | 154 |
| ***RORA*** | Homo sapiens RAR related orphan receptor A | AAATCGCATCTGGAAACCTG | TTGGCAAACTCCACCACATA | NM_134261.3 | 173 |
|  |  |  |  | NM_134260.3 |  |
|  |  |  |  | NM_002943.4 |  |
|  |  |  |  | NM_134262.3 |  |
